# Tailored synthesis and morphological analysis of Mo_2_CT_*x*_ and Ti_3_C_2_T_*x*_ MXenes: a study on multilayered and delaminated architectures

**DOI:** 10.1039/d5na00669d

**Published:** 2025-11-03

**Authors:** Vasanth Magesh, Raji Atchudan, Sandeep Arya, Surendra H. Mahadevegowda, Ashok K. Sundramoorthy

**Affiliations:** a Department of Prosthodontics and Materials Science, Saveetha Dental College and Hospitals, Saveetha Institute of Medical and Technical Sciences Chennai 600077 Tamil Nadu India ashok.sundramoorthy@gmail.com; b School of Chemical Engineering, Yeungnam University Gyeongsan 38541 Republic of Korea; c Department of Physics, University of Jammu Jammu 180006 Jammu and Kashmir India; d Department of Chemistry, School of Sciences, National Institute of Technology Andhra Pradesh Tadepalligudem 534101 Andhra Pradesh India

## Abstract

MXenes, a family of two-dimensional transition metal carbides, nitrides, and carbonitrides, display high conductivity, large surface area, hydrophilicity, and biocompatibility. This work demonstrates a comparative scalable synthesis of multilayer (m-MXene) and delaminated (d-MXene) Mo_2_CT_*x*_ and Ti_3_C_2_T_*x*_ from their MAX phases (Mo_2_Ga_2_C and Ti_3_AlC_2_) using optimized HCl/HF/DI water etchants (ratios of 6 : 3 : 1 for Mo_2_Ga_2_C and 6 : 1 : 3 for Ti_3_AlC_2_) *via* an LiCl-assisted delamination strategy. Significant interlayer expansion is confirmed by XRD (002) peak shifts (from 9.81° to 8.03° for Mo_2_CT_*x*_ and from 9.51° to 8.80° for Ti_3_C_2_T_*x*_), while their lateral sizes reached 2–5 µm for Ti_3_C_2_T_*x*_ and 0.5–1 µm for Mo_2_CT_*x*_. The UV-Vis spectra showed characteristic absorption peaks at 209, 230, 295, and 568 nm for Mo_2_CT_*x*_ and at 263, 325, and 798 nm for Ti_3_C_2_T_*x*_, confirming their delamination and distinctive electronic structure. Thorough structural and compositional characterizations (UV-Vis spectroscopy, XRD, HR-SEM, EDS, Raman spectroscopy, and FTIR spectroscopy) verified the successful synthesis of MXenes. This study provides the first direct systematic comparison of Mo_2_CT_*x*_ and Ti_3_C_2_T_*x*_ MXenes, establishing benchmarks for the scalable production and applications of MXenes.

## Abstract 2

The study shows MAX phases converting to delaminated MXene. HF etching removes the A-layer, forming multilayer MXene with surface terminations. Li^+^ intercalation expands layers, and delamination yields stable mono/few-layer MXene.

## Graphical abstract

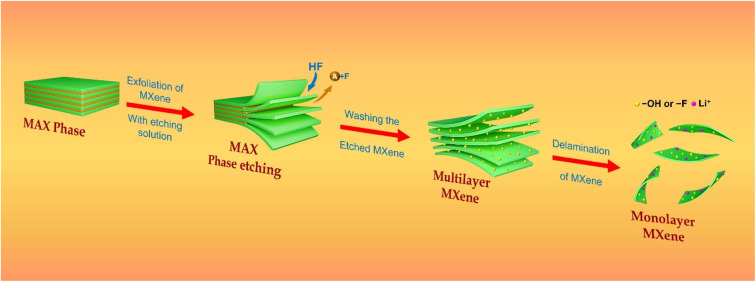

## 1 Introduction

MXenes are two-dimensional (2-D) transition metal carbides, nitrides, and carbonitrides with the general formula of M_*n*+1_X_*n*_T_*x*_. In this formula, “M” represents an early transition metal (such as Ti, Mo, V, Sc, Nb, and Cr), “X” is carbon and/or nitrogen, “*n*” is an integer from 1 to 4 indicating the layer thickness, and “T_*x*_” represents surface functional groups (–F, –O, and –OH) introduced during the wet chemical etching process, where “A” is typically a group 13 or 14 element, except boron (B) and carbon (C).^1,2^ MXenes have gained global research interest for their potential in energy storage, photovoltaics, nanoelectronics, and optoelectronics,^3,4^ motivating fundamental studies on their synthesis, structure, and surface chemistry. Both stacked multilayer and single-layer delaminated MXenes have attracted tremendous attention in many fields including supercapacitors, sensors, EMI shielding, actuators, field-effect transistors, photothermal therapy, and cancer treatment.^5–12^ Their unique properties, including high electrical conductivity,^13^ metallic nature,^14^ hydrophilicity,^15^ biocompatibility,^16,17^ and large surface area, make MXenes highly valuable across these research fields.^18^ Recent publications have highlighted Ti_3_C_2_T_*x*_, Mo_2_CT_*x*_, and other related MXenes and their applications in adsorption,^19^ antimicrobial activity,^20^ environmental remediation,^21^ water treatment and pollution control.^21,22^ To date, over 62 distinct MXenes have been synthesized experimentally, and many others have been theoretically examined.^23–25^ However, the majority of research to date has focused on Ti_3_C_2_T_*x*_.^26,27^

Various synthesis methods have been developed to etch the MAX phase for producing multilayer MXenes (m-MXenes) and to utilize intercalating agents for delaminating m-MXenes into few-layer or monolayer MXenes (d-MXenes).^28^ The etching and delamination processes significantly influence the shape, size, morphology, and surface chemistry of MXenes, which in turn affect their processing and performance.^14,29–31^ Wet chemical etching is the most commonly used method for preparing MXenes. In this approach, immersing MAX phases in concentrated hydrofluoric acid (HF) leads to the separation of layers and the introduction of –OH and –F terminal groups.^32^ During this wet etching, ‘A’-layer is gradually removed to produce m-MXenes, while organic agents (*e.g.*, TBAOH and DMSO) or inorganic compounds (*e.g.*, cationic surfactants) are used to delaminate m-MXenes into d-MXenes.^28^ However, HF-etched MXenes often exhibit small lateral sizes and structural defects.^33,34^ To address these issues, a minimally intensive layer delamination (MILD) method was adopted to produce high-quality MXenes, which was based on *in situ* HF generated using LiF, HCl and bifluoride-based etchants (NH_4_HF_2_, NaHF_2_, and KHF_2_).^35–39^ These methods have their own strengths and limitations. MILD etching requires extended durations at room temperature (RT) or shorter times at elevated temperatures and typically involves organic intercalation or ultrasonication for successful delamination. In contrast, the *in situ* HF method produces clay-like aggregated m-MXenes, and delamination into d-MXenes can be achieved using deionized (DI) water by a simple hand-shaking liquid-assisted process (no need for organic intercalation or ultrasonication).^28,40^ However, the *in situ* formation of HF leads to a slower etching rate and sometimes incomplete removal of the A-layer, which may leave residual A-site elements in the MXene structure due to its less aggressive nature compared to concentrated HF. Therefore, longer etching durations are required, which can affect the overall quality and yield of the resulting MXene.^41^ Additionally, the etching process can generate unwanted byproducts such as salts (*e.g.*, LiCl and GaF_3_) or gel-like residues during the washing process, which are difficult to remove completely and may also interfere with the delamination process and the final application of MXenes.^42–44^ Bifluoride-based etching methods require additional steps to remove the residual inorganic cations after the etching process, especially when intended for specific applications.^45,46^ Furthermore, Mathis *et al.* emphasized that the production of high-quality m-MXenes and d-MXenes depends not only on optimized etching methods but also on the quality of the starting MAX phase.^47^ They had demonstrated that synthesizing Ti_3_AlC_2_ with an excess of aluminum enhances the structural integrity and morphology of MAX phase grains. This approach resulted in high-quality Al–Ti_3_C_2_T_*x*_ MXene nanosheets with superior chemical stability, allowing storage for up to 10 months without significant oxidation or degradation.^47^ This study highlights the importance of optimizing both the MAX phase synthesis protocols and etching processes to achieve consistent and reliable MXene production.

Despite notable progress in MXene synthesis methodologies, significant research gaps persist, particularly in conducting systematic comparative studies among different MXene types and optimizing synthesis protocols for less-explored families such as Mo_2_CT_*x*_. Most existing studies focus on individual MXenes, which restricts our understanding of how synthesis parameters should be customized for distinct MAX phase precursors with diverse chemical compositions. Moreover, the correlation between precursor properties and the resulting d-MXene morphology remains poorly elucidated.

To bridge these gaps, the present study reports the first comprehensive comparative synthesis and characterization of Mo_2_CT_*x*_ and Ti_3_C_2_T_*x*_ MXenes under identical experimental conditions. The novelty of this work is fourfold: (1) optimization of etchant composition (HCl : HF : DI water), determining ideal ratios (6 : 3 : 1 for Mo_2_Ga_2_C and 6 : 1 : 3 for Ti_3_AlC_2_) based on the intrinsic chemical differences between Mo–Ga and Ti–Al bonding in their respective MAX phases; (2) detailed morphological and structural comparison of Mo- and Ti-based MXenes throughout the synthesis pathway (MAX → m-MXene → d-MXene); (3) successful fabrication of d-MXenes *via* LiCl intercalation alone, removing the need for organic intercalants, ultrasonication, or other aggressive delamination treatments; and (4) systematic evaluation of precursor influence on final d-MXene lateral dimensions, revealing that Ti_3_C_2_T_*x*_ MXenes exhibit 2–5 µm lateral sizes, whereas Mo_2_CT_*x*_ MXenes achieve 0.5–1 µm when subjected to optimized HCl/HF/DI water etching followed by LiCl-assisted delamination at room temperature. This integrated approach effectively promotes interlayer expansion and enables direct side-by-side assessment of Mo- and Ti-based MXenes, offering valuable insights into how etching and delamination conditions affect MXene structural integrity.

Overall, this study establishes the first unified comparative framework that links synthesis parameters, structural evolution, and morphological characteristics across different MXene families. The findings provide critical benchmarks for optimizing MXene synthesis and deepen the understanding of how MAX phase chemistry dictates the properties of derived 2D MXenes. This comparative methodology is anticipated to advance the rational design of synthesis strategies for emerging MXene compositions, facilitating more predictable control over their structure and performance for diverse applications.

## 2 Experimental

### 2.1 Chemicals and reagents

Titanium aluminum carbide (Ti_3_AlC_2_) and molybdenum gallium carbide (Mo_2_Ga_2_C) MAX phase powders were purchased from Aritech Chemazone, India. Hydrochloric acid (HCl, 35%) and lithium chloride (LiCl, 99%) were obtained from Sigma-Aldrich, India. Hydrofluoric acid (HF, 48%) was sourced from NICE Chemicals, India. All chemicals were used as received, without further purification. Common laboratory-grade reagents were employed as per standard protocols. Distilled water (DI water) with a resistivity of 18.2 MΩ·cm was supplied using a Millipore system.

### 2.2 Preparation of Mo_2_CT_*x*_ m-MXenes and d-MXenes

**Fig. 1:**
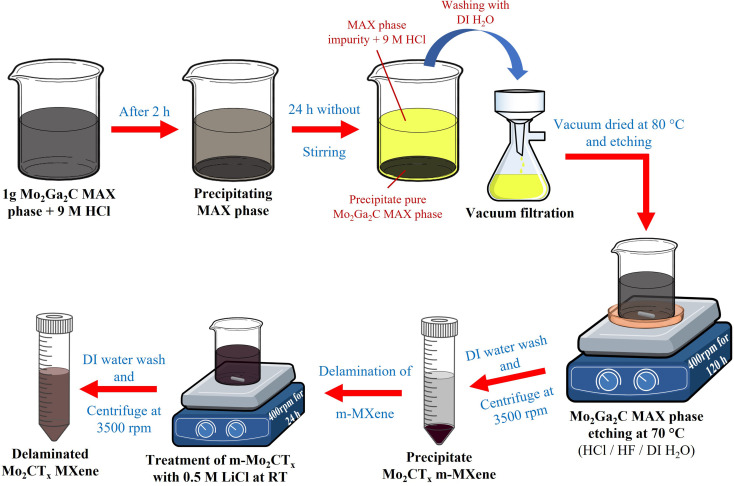
Schematic of the etching and delamination process of Mo_2_CT_*x*_ from the Mo_2_Ga_2_C MAX phase. The diagram shows the selective etching of Ga layers followed by LiCl-assisted delamination, yielding multilayer and delaminated Mo_2_CT_*x*_ sheets.

To prepare Mo_2_CT_*x*_ m-MXenes, the Mo_2_Ga_2_C MAX phase powder was pretreated to remove impurities (unreacted gallium (Ga), molybdenum (Mo), and carbon (C) during MAX phase synthesis), and then wet etching was performed. For the removal of impurities, 1 g of Mo_2_Ga_2_C MAX phase powder was pretreated with 30 mL of 9 M HCl for 24 h.^47^ During this process, the solution turned yellow after 10 h and became completely golden yellow within 24 h (Fig. 1 and S1A). The treated powder was vacuum-filtered, thoroughly rinsed with DI water, and dried at 80 °C for 24 h in a vacuum oven under −25 inHg pressure, resulting in a greenish-gray powder with a flour-like texture (Fig. S2A). Next, 0.5 g of the dried powder was etched in 30 mL of a solution comprising 12 M HCl, 48% HF, and DI water in a ratio of 6 : 3 : 1.^47^ The etching process was conducted at 70 °C ± 3 °C in an oil bath with continuous stirring at 400 rpm for 120 h. The resulting etched mixture was washed multiple times by centrifugation at 3500 rpm for 5 min per cycle using 30 mL of DI water. Washing process was continued until the supernatant achieved a pH between 6 and 7, which was typically reached after the fifth wash.

For Mo_2_CT_*x*_ d-MXene preparation, the centrifuged precipitate (m-Mo_2_CT_*x*_) was added to 50 mL of 0.5 M LiCl and stirred at 400 rpm at RT for 24 h.^47^ The mixture was washed by centrifugation at 3500 rpm for 5 min to remove unreacted LiCl. By the fourth wash, the supernatant became slightly transparent with a brown tint, indicating the successful removal of excess LiCl. After the fourth wash, N_2_-purged DI water was added to the precipitate, hand-shaken for 2 min and centrifuged at 3500 rpm for 30 min. The resulting supernatant appeared as a transparent brownish-green solution (Fig. S3A), confirming the delamination of Mo_2_CT_*x*_ MXenes. This process was repeated three times, and the collected transparent brownish-green supernatant containing the d-MXene was stored separately. After three repetitions, the remaining precipitate was re-dispersed and stored in tightly sealed PTFE bottles at 4 °C.

The product yields at each synthesis stage are summarized as follows: For Mo_2_CT_*x*_, starting from 500 mg of Mo_2_Ga_2_C MAX precursor, approximately 305 mg of multilayer MXenes were obtained after acid etching, corresponding to a yield of ∼61%. Subsequent delamination produced about 120 mg of delaminated MXenes, equivalent to ∼24% of the initial MAX precursor. Some material loss is expected during repeated washing steps following etching and LiCl treatment. Only the delaminated monolayer and few-layer MXene sheets remain suspended in the supernatant after sedimentation, representing the actual yield of the delaminated product. These variations in yield are attributed to the intrinsic structure of the MAX phase and the efficiency of the etching process.

### 2.3 Preparation of Ti_3_C_2_T_*x*_ m-MXenes and d-MXenes

**Fig. 2:**
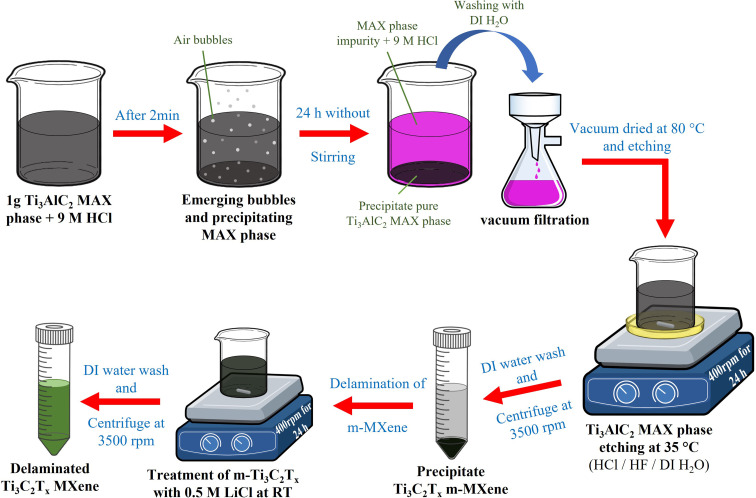
Schematic of the etching and delamination process of Ti_3_C_2_T_*x*_ from the Ti_3_AlC_2_ MAX phase. The scheme depicts selective Al removal using an acid mixture and subsequent LiCl-assisted delamination, producing multilayer and delaminated Ti_3_C_2_T_*x*_ sheets.

The Ti_3_C_2_T_*x*_ MXene was synthesized by following a similar procedure to that used for Mo_2_CT_*x*_ with slight changes. First, 1 g of Ti_3_AlC_2_ powder was immersed in 30 mL of 9 M HCl for 24 h at RT without stirring to remove impurities (unreacted titanium (Ti), aluminum (Al), and C during MAX phase synthesis).^47^ In the beginning, (first 2 min), the air bubbles began to emerge from the powder in the HCl solution (likely hydrogen gas [2Al_(s)_ + 6HCl_(aq)_ → 2AlCl_3(aq)_ + 3H_2(g)_]) (Fig. 2). After 10 h, the solution's color shifted to a pinkish–purple hue, and later it turned completely pinkish–purple after 24 h (Fig. S1B).^28^ The treated powder was then filtered using a vacuum filtration system and rinsed several times with DI water to remove residual HCl. Subsequently, the powder was dried at 80 °C for 24 h in a vacuum oven under a pressure of −25 inHg, resulting in a blackish-gray powder with a texture resembling that of fine black-sand (Fig. S2B). Next, 0.5 g of the dried powder was etched in 30 mL of an etching solution composed of 12 M HCl, 48% HF, and DI water in a ratio of 6 : 1 : 3.^47^ The etching process was conducted with stirring at 400 rpm for 24 h at a controlled temperature of 35 ± 2 °C using an oil bath. The resulting mixture was washed repeatedly by centrifugation at 3500 rpm for 5 min per cycle with 30 mL of DI water until the supernatant achieved a pH of 6 or higher. In this procedure, the pH of the supernatant was stabilized between 6 and 7 after the sixth wash.

For Ti_3_C_2_T_*x*_ d-MXene preparation, the centrifuged precipitate of m-MXenes was added to 50 mL of 0.5 M LiCl solution and stirred at 400 rpm at RT for 24 h.^47^ The mixture was then washed with DI water by hand-shaking for 1 min, followed by centrifugation at 3500 rpm for 5 min to remove unreacted LiCl. After the third wash, the supernatant became slightly transparent with a greenish tint, indicating the successful removal of unreacted LiCl. Thereafter, N_2_-purged DI water was added to the precipitate, hand-shaken for 2 min, and centrifuged at 3500 rpm for 30 min. Finally, the supernatant appeared as a transparent green solution (Fig. S3B), confirming the delamination of Ti_3_C_2_T_*x*_ MXenes. This process was repeated three times, and the collected transparent green supernatant containing the d-MXene was stored separately. After three repetitions, the remaining precipitate was re-dispersed and stored in tightly sealed PTFE bottles at 4 °C.

The mass yields at each synthesis stage are summarized as follows: for Ti_3_C_2_T_*x*_, acid etching of 500 mg of Ti_3_AlC_2_ MAX precursor produced approximately 360 mg of multilayer MXenes, corresponding to a yield of ∼72%. Subsequent delamination yielded around 155 mg of delaminated MXenes, equivalent to ∼31% of the initial MAX material. Minor material loss occurs during the repeated washing steps following etching and LiCl treatment. The true yield of the delaminated product corresponds to the monolayer and few-layer MXene sheets that remain suspended in the supernatant after sedimentation. These yield variations are influenced by the intrinsic MAX phase structure and the efficiency of the etching process.

### 2.4 Instrumentation

The absorption spectra of the Ti_3_C_2_T_*x*_ and Mo_2_CT_*x*_ d-MXenes were examined by UV-Visible spectroscopy (UV-Vis, LAMBDA 365+, PerkinElmer, USA). A D8-Advance XRD (BRUKER, Billerica, MA, USA) was used for recording the X-ray diffraction (XRD) data. The surface structure and morphology of the Ti_3_C_2_T_*x*_ and Mo_2_CT_*x*_ d-MXenes were investigated using a high-resolution scanning electron microscope (HR-SEM, Quanta FEG 200F, FEI, USA). Energy-dispersive X-ray spectrum (EDS) data were obtained using an XPLORE-30 (Oxford, UK). Raman spectra were acquired using an XPLORA plus (HORIBA, Kyoto, Japan) with 532 nm laser excitation. Spectrum-II (PerkinElmer, USA) equipment was used to record the Fourier transform infrared (FTIR) spectra. All characterizations (except UV-Vis) were performed using vacuum-dried powder samples of the MAX phases, m-MXenes, and d-MXenes.

## 3 Results and discussion

### 3.1 UV-visible spectra of Mo_2_CT_*x*_ and Ti_3_C_2_T_*x*_ d-MXenes

**Fig. 3:**
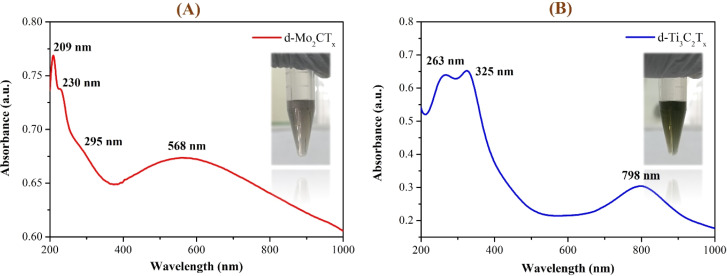
UV-visible absorption spectra of delaminated MXene suspensions: characteristic absorption peaks of (A) Mo_2_CT_*x*_ and (B) Ti_3_C_2_T_*x*_, highlighting their distinct electronic structures. Insets: photographs of the d-MXene dispersed solutions, indicating colloidal stability.

The UV-Vis spectra of Mo_2_CT_*x*_ and Ti_3_C_2_T_*x*_ d-MXenes reveal distinct absorbance peaks for each material. Mo_2_CT_*x*_ exhibits peaks at 209 nm, 230 nm, 295 nm, and 568 nm (Fig. 3A), whereas Ti_3_C_2_T_*x*_ shows peaks at 263 nm, 325 nm, and 798 nm (Fig. 3B).^48^ The absorption peaks at 209 nm and 263 nm are attributed to π → π* transitions within the Mo–C and Ti–C framework, reflecting strong electronic interactions in their carbon structures.^49^ The absorption peak at 230 nm is also attributed to the π → π* transitions in the Mo–C framework.

The dual peaks observed at 209 nm and 230 nm for Mo_2_CT_*x*_ d-MXenes with the same transition are probably due to differences in bonding environments and the influence of surface terminations such as –OH, –F, or =O groups. The peaks at 295 nm (Mo_2_CT_*x*_) and 325 nm (Ti_3_C_2_T_*x*_) are probably attributed to n → π* transitions, involving lone pairs of electrons on oxygen or other surface groups, interacting with the carbon π-system. These transitions may also be influenced by the surface terminations of MXenes.^49^ In the visible region, a broad peak at 568 nm (for Mo_2_CT_*x*_) suggests d–d electronic transitions characteristic of molybdenum atoms. Similarly, the absorbance at 798 nm in the near-infrared region for Ti_3_C_2_T_*x*_ indicates d–d transitions typical of titanium atoms.^28,50^ The absorption peaks observed in the visible and near-infrared regions reflect significant electronic interactions within the Mo_2_CT_*x*_ and Ti_3_C_2_T_*x*_ d-MXene structures. These observations indicated the charge transfer between metal centers and ligands, influenced by surface terminations.^51,52^ Additionally, the broad absorbance features may arise from plasmonic-like collective oscillations of delocalized electrons, as suggested in earlier MXene studies.^53,54^

UV-Vis spectroscopy validated the optical properties and colloidal stability of both Mo_2_CT_*x*_ and Ti_3_C_2_T_*x*_ MXenes. The observed electronic transitions in their UV-Vis spectra are consistent with the characteristic features reported by Alhabeb *et al.* (2017), Mathis *et al.* (2021) and Sunderiya *et al.* (2024).^28,47,55^ These results confirm that the optical transitions and dispersion achieved in this study are in line with other earlier studies, affirming successful synthesis and delamination of both Mo- and Ti-based MXenes for advanced applications. To further support these results, the oxidation stability of delaminated Mo_2_CT_*x*_ and Ti_3_C_2_T_*x*_ MXene dispersions was evaluated by monitoring their colloidal stability in DI water over 32 days under ambient conditions (Fig. S4). Both dispersions maintained a stable, highly dispersed state with no visible sedimentation or color change throughout this period. The minimal sedimentation observed after 32 days indicated excellent resistance to oxidation and prolonged colloidal stability for both MXene types. While these observations demonstrated good stability over 32 days, the longer-term oxidation stability was not assessed. Moreover, the 32-day data suggested that the as-prepared MXenes have strong resistance to oxidation and sustained dispersion quality, supporting the retention of electronic and optical transitions, as reflected by the UV-Vis spectra.

### 3.2 XRD analysis of Mo_2_Ga_2_C and Ti_3_AlC_2_ systems

**Fig. 4:**
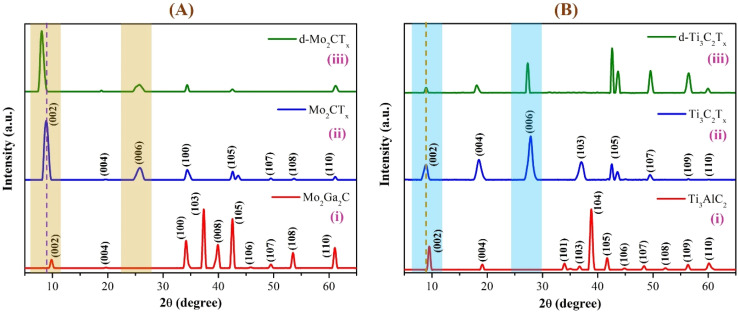
XRD patterns of (A) Mo_2_Ga_2_C and (B) Ti_3_AlC_2_ systems showing (i) MAX phase, (ii) m-MXene, and (iii) d-MXene forms. The graphs highlighted in yellow and blue indicate that etching induces a shift in the (002) peak toward lower angles and the appearance of the (006) peak in m-MXenes. Delamination further shifts the (002) peak due to Li^+^ intercalation and interlayer expansion.

The XRD results of the Mo_2_Ga_2_C and Ti_3_AlC_2_ MAX phases, along with their corresponding m-MXene and d-MXene forms, are presented in Fig. 4. The XRD pattern of the pure Mo_2_Ga_2_C MAX phase (Fig. 4A(i), red curve) exhibits diffraction peaks at 9.81° (002), 19.68° (004), 34.16° (100), 37.35° (103), 39.91° (008), 42.56° (105), 45.86° (106), 49.48° (107), 53.51° (108), and 61.06° (110).^56,57^ After etching Ga from Mo_2_Ga_2_C, significant changes in crystallinity and structure were observed, as shown in the m-MXene XRD pattern (Fig. 4A(ii), blue curve). The (002) peak at 9.81° shifted slightly to a lower angle of 8.86°, indicating an increased *d*-spacing due to the expanded interlayer gap. A new peak appeared at 25.77°, corresponding to the (006) reflection. This new peak is a characteristic feature of Mo_2_CT_*x*_ MXenes, signifying successful etching and the formation of the layered MXene structure.^56,58^ In the Mo_2_Ga_2_C MAX phase, the compact structure and the presence of Ga layers suppress the periodicity required for the (006) reflection. After Ga is eliminated, the Mo_2_C layers undergo rearrangement, establishing a new periodicity along the *c*-axis and resulting in the emergence of the (006) peak.^59^ The disappearance of the (103) and (008) peaks at 37.35° and 39.94°, respectively, further confirms the successful removal of Ga. Additionally, the (100) peak, originally located at 34.16°, shifted slightly to a higher angle of 34.38° after Ga removal. This shift can be attributed to a slight contraction in the lattice structure within the *ab*-plane (*a* and *b* axes).^60^ The removal of Ga reduces interlayer interactions, leading to a decrease in lattice parameters and causing the diffraction angle of the (100) plane to increase, as explained by Bragg's law.^61^ These structural changes reflect the successful etching of Ga and the subsequent formation of the Mo_2_CT_*x*_ MXene. The XRD pattern of the Mo_2_CT_*x*_ d-MXene (Fig. 4A(iii), green curve) reveals a reduction in the intensity of most peaks, except for the (002) peak. Notably, the (002) peak is further shifted to a lower angle of 8.03°. This shift is attributed to the intercalation of Li^+^ ions during the delamination process, which increases the interlayer spacing between the sheets.

The XRD pattern of the pure Ti_3_AlC_2_ MAX phase (Fig. 4B(i), red curve, JCPDS No. 52-0875) exhibits diffraction peaks at 9.51° (002), 19.14° (004), 33.98° (101), 36.70° (103), 38.85° (104), 41.71° (105), 44.82° (106), 48.38° (107), 52.25° (108), 56.31° (109), and 60.17° (110).^62–64^ After etching aluminum (Al) from Ti_3_AlC_2_, the crystallinity and structure underwent significant changes, as evident from the m-MXene XRD pattern (Fig. 4B(ii), blue curve). The (002) and (004) peaks of Ti_3_AlC_2_, originally found at 9.51° and 19.14°, were shifted to lower angles of 8.92° and 18.48°, respectively.

This indicated an increased *d*-spacing due to the expanded interlayer gap. A new peak at 27.86°, corresponding to the (006) reflection, emerged as a characteristic feature of Ti_3_C_2_T_*x*_ MXene, signifying successful etching and the formation of the layered MXene structure.^65–67^ In the Ti_3_AlC_2_ MAX phase, the compact structure and presence of Al layers inhibit the periodicity required for the (006) reflection. Upon Al removal, the Ti_3_C_2_ layers rearrange and create a new periodicity along the *c*-axis, which gives rise to the (006) peak.^68^ Additionally, the absence of the (104) peak at 38.85°, originally present in the MAX phase, further corroborates the successful removal of Al.^69^ The Ti_3_C_2_T_*x*_ d-MXene XRD pattern (Fig. 4B(iii), green curve) reveals a reduction in the intensity of the (002), (004), and (006) peaks, which have slightly shifted to lower angles of 8.80°, 18.25°, and 27.32°, respectively. These shifts result from the intercalation of Li^+^ ions, which increase the *d*-spacing between the sheets during the delamination process.^70,71^ Additionally, delaminated MXene layers align preferentially with their basal planes parallel to the substrate or solvent interface, altering the XRD patterns. In Mo_2_CT_*x*_ d-MXenes, this alignment enhances basal reflections ((002) and (006)) while reducing the intensity of the (100) peak, confirming successful delamination. In the Ti_3_C_2_T_*x*_ d-MXene, the opposite trend is observed, with basal plane reflections being reduced and the (103) peak diminished or disappeared, indicating successful delamination. The combined effects of peak shifts, reduced peak intensities, and the disappearance of specific peaks demonstrated the successful exfoliation of both Mo_2_CT_*x*_ and Ti_3_C_2_T_*x*_ MXenes into monolayer or few-layer delaminated sheets. The observed (002) diffraction peak shift for Ti_3_C_2_T_*x*_ (from 9.51° in the MAX phase to 8.80° in the d-MXene) is consistent with the values reported by Naguib *et al.* and Ghidiu *et al.* (2014), confirming a significant increase in interlayer spacing upon delamination and Li^+^ intercalation.^40,62^ Similarly, for Mo_2_CT_*x*_, the (002) peak shift aligns with the findings of Gupta *et al.* (2023) and Pazniak *et al.* (2021), which also attribute to the expansion in *d*-spacing to Li^+^ insertion during the delamination process.^56,57^ These comparable results validated the efficiency of the tailored synthesis protocol employed in this study for achieving effective intercalation and exfoliation.

To quantify the structural changes, we calculated the (002) interlayer spacing from Bragg's law (eqn (1) and (2)) using Cu Kα radiation (*λ* = 1.5406 Å) (Table 1). The Mo-system shows an increase in d (002) from 9.01 Å (Mo_2_Ga_2_C MAX) to 9.97 Å (m-Mo_2_CT_*x*_) and to 11.00 Å after delamination (d-Mo_2_CT_*x*_), corresponding to +10.70% and +22.12% increases, respectively. The Ti-system increases from 9.29 Å (Ti_3_AlC_2_ MAX) to 9.91 Å (m-Ti_3_C_2_T_*x*_) and to 10.04 Å (d-Ti_3_C_2_T_*x*_), *i.e.*, +6.60% and +8.06%, respectively. These quantitative changes support the interpretation that Li^+^ intercalation and delamination substantially increase the interlayer separation, and that the Mo-system undergoes a larger relative expansion than the Ti-system under our etching/delamination conditions.

1 *nλ* = 2*d* sin *θ*

2
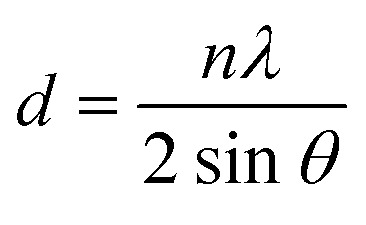


**Table 1:** Interlayer spacings (d (002)) and % changes of Mo_2_CT_*x*_ and Ti_3_C_2_T_*x*_ compared with their parent MAX phases using Bragg's law

System	Phase	2*θ* (°) (current work)	d (002) (Å)	% Change *vs.* MAX
Mo_2_Ga_2_C → Mo_2_CT_*x*_	MAX (002)	9.81	9.009 Å	—
	m-MXene (002)	8.86	9.973 Å	10.70%
	d-MXene (002)	8.03	11.002 Å	22.12%
Ti_3_AlC_2_ → Ti_3_C_2_T_*x*_	MAX (002)	9.51	9.292 Å	—
	m-MXene (002)	8.92	9.906 Å	6.60%
	d-MXene (002)	8.8	10.041 Å	8.06%

### 3.3 HR-SEM and EDS analysis of Mo_2_Ga_2_C and Ti_3_AlC_2_ systems

**Fig. 5:**
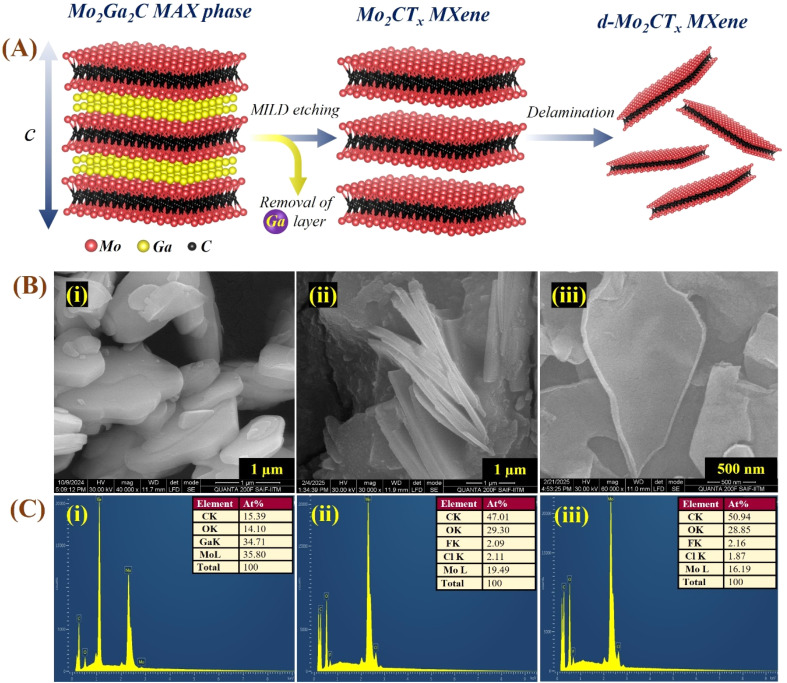
(A) Molecular models, illustrating the Mo_2_Ga_2_C MAX phase, selective Ga removal to form m-MXenes (Mo_2_CT_*x*_), and LiCl-assisted delamination yielding mono- and few-layer sheets. (B) SEM images, indicating the morphological changes: (i) white-stone-like Mo_2_Ga_2_C MAX (∼2–4 µm), (ii) stacked multilayer Mo_2_CT_*x*_ (∼1–2 µm), and (iii) exfoliated, bent-edge delaminated Mo_2_CT_*x*_ sheets (∼0.5–1 µm). (C) EDS spectra, showing the corresponding compositional changes: (i) Mo, Ga, C, O in MAX; (ii) strong Ga reduction and appearance of F/Cl terminations in m-MXenes; and (iii) enhanced surface functionalization in d-MXenes.

**Fig. 6:**
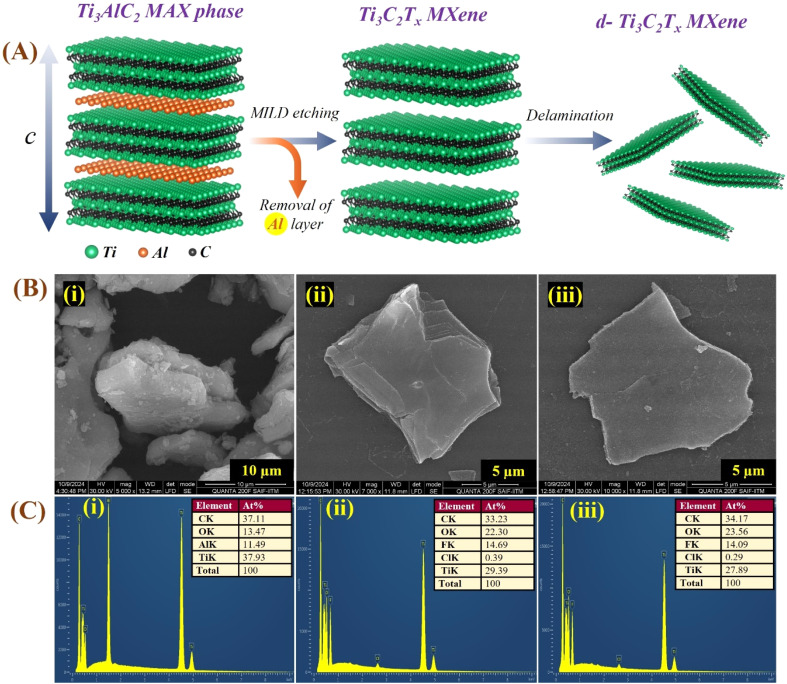
(A) Molecular models, illustrating the transformation of Ti_3_AlC_2_ MAX into multilayer Ti_3_C_2_T_*x*_ (m-MXene) after selective Al removal, followed by LiCl-assisted delamination yielding mono- and few-layer sheets. (B) SEM images, indicating the morphological changes: (i) dense rock-like Ti_3_AlC_2_ MAX (∼10–15 µm), (ii) stacked multilayer Ti_3_C_2_T_*x*_ (∼5–10 µm), and (iii) exfoliated, sharp-edge delaminated sheets (∼1–2 µm). (C) EDS spectra, showing the elemental compositional changes: (i) Ti, Al, C, and O in MAX; (ii) significant Al removal and appearance of F/Cl terminations in m-MXenes; and (iii) enhanced surface functionalization in d-MXenes.

The surface morphology and elemental composition of Mo_2_Ga_2_C and Ti_3_AlC_2_ MAX phases, m-MXenes, and d-MXenes were analyzed by HR-SEM imaging and EDS, as illustrated in Fig. 5 and 6. The molecular structures of the Mo_2_Ga_2_C and Ti_3_AlC_2_ MAX phases, along with the etched m-MXenes and d-MXenes, are depicted in Fig. 5A and 6A. The HR-SEM images of the Mo_2_Ga_2_C MAX phase revealed a white-stone-like morphology characteristic of Mo-MAX phases with well-defined stacked grains (Fig. 5B(i)). After the etching process to produce Mo_2_CT_*x*_ m-MXenes, the HR-SEM images revealed significant exfoliation of Ga layers, resulting in a loosely packed layered morphology (Fig. 5B(ii)). In the proposed etching method, the Mo_2_Ga_2_C MAX phase did not undergo vigorous etching as it would with highly concentrated HF. The slow removal of the A-layer and gradual release of H_2_ during etching led to the formation of this type of structure. The d-Mo_2_CT_*x*_ MXene demonstrated further delamination, with monolayer sheets, confirming the successful delamination of Mo_2_CT_*x*_MXenes (Fig. 5B(iii)). The delaminated MXene sheets exhibited slightly bent edges and a smooth surface morphology.

Similarly, the HR-SEM images of the Ti_3_AlC_2_ MAX phase display its characteristic rock-like layered morphology, with individual particles measuring approximately 10–15 µm and exhibiting distinctly stacked grains (Fig. 6B(i)). Upon etching to form the multilayer Ti_3_C_2_T_*x*_ (m-MXene), the morphology transforms into a layered structure (Fig. 6B(ii)), where the stacked layers, oriented perpendicular to the imaging plane, clearly reveal the material's multi-layered nature.^72,73^ Subsequent delamination leads to the formation of Ti_3_C_2_T_*x*_ d-MXenes, consisting of completely separated, monolayer MXene sheets (Fig. 6B(iii)).

The d-MXene sheets of Ti_3_C_2_T_*x*_ exhibited sharp edges and a smooth surface, in contrast to the bent edges observed in Mo_2_CT_*x*_ d-MXenes. This difference can be attributed to the structural composition of the MXenes: Ti_3_C_2_T_*x*_ has three layers of titanium and two layers of carbon in each sheet, whereas Mo_2_CT_*x*_ comprises two layers of molybdenum and one layer of carbon, making the Mo_2_CT_*x*_ sheets more prone to bending and uneven edges compared to the sharper edges of Ti_3_C_2_T_*x*_ MXenes. The HR-SEM images showed larger lateral sizes for the Ti_3_AlC_2_ MAX phase (5–10 µm) compared to the Mo_2_Ga_2_C MAX phase (1–2 µm). After delamination, the lateral size of the Ti_3_C_2_T_*x*_ MXene observed in this study (2–5 µm) is slightly larger but broadly consistent with the atomic-scale characterization reported by Pazniak *et al.* (2019), who produced Ti_3_C_2_T_*x*_ flakes with an average lateral size close to 1–2 µm with uniform morphology using self-propagation high-temperature synthesis (SHS) and grinding to produce Ti_3_AlC_2_ MAX phases.^74^ For Mo_2_CT_*x*_ MXenes, the observed lateral sizes (0.5–1 µm) are comparable to those reported by Guo *et al.*(2020),^75^ who found it difficult to obtain Mo_2_CT_*x*_ flakes larger than 1 µm regardless of etching technique, where most flakes being sub-micrometer in size. The lateral sheet size distributions of the exfoliated Mo_2_CT_*x*_ and Ti_3_C_2_T_*x*_ MXenes were quantified using the ImageJ software from the HR-SEM images. The size measurements were plotted as histograms and fitted with a normal (Gaussian) distribution using the Origin software (shown in Fig. S5). This analysis revealed a notable difference in mean lateral sheet sizes: approximately 620 nm for Mo_2_CT_*x*_ and 3.7 µm for Ti_3_C_2_T_*x*_. These findings confirmed that both of the synthesis routes and the quality of the precursor MAX phase play crucial roles in determining MXene sheet morphology and size.

The EDS spectra were used to confirm the formation of Mo_2_CT_*x*_ by analyzing the atomic weight percentages of elemental compositions in the MAX phase, m-MXene, and d-MXene. The MAX phase (Mo_2_Ga_2_C) showed the presence of Mo (35.80%), Ga (34.71%), C (15.39%), and O (14.10%) (Fig. 5C(i)). For Mo_2_CTx m-MXenes, the EDS spectrum confirmed the presence of Mo (19.49%), C (47.01%), and O (29.30%) (Fig. 5C(ii)). Moreover, fluorine (F, 2.09%) and chlorine (Cl, 2.11%) were detected in the EDS spectrum of the m-MXene, originating from the etchant used. In the Mo_2_CT_*x*_ d-MXene, the EDS analysis confirmed the presence of Mo (16.19%), C (50.94%), and O (28.85%) (Fig. 5C(iii)).

Similarly, the EDS spectrum of the Ti_3_AlC_2_ MAX phase (Fig. 6C(i)) confirmed its elemental composition, with Ti (37.93%), Al (11.49%), C (37.11%), and O (13.47%). In the m-MXene (Ti_3_C_2_T_*x*_) spectrum (Fig. 6C(ii)), the removal of Al is evident, along with increased ‘O’ and ‘F’ contents, attributed to surface terminations. The elemental composition of m-MXenes includes Ti (29.39%), C (33.23%), O (22.30%), F (14.69%), and Cl (0.39%). For d-MXenes (Fig. 6C(iii)), the spectrum highlighted the presence of Ti (27.89%), C (34.17%), O (23.56%), F (14.09%), and Cl (0.29%), with a significantly reduced Al content, further confirming successful etching and delamination processes. The EDS analysis of both Mo_2_CT_*x*_ and Ti_3_C_2_T_*x*_ d-MXenes showed elements with weight percentages, which are well consistent with the values reported by Guo *et al.* (2020), Wei *et al.* (2023), Yan *et al.* (2019) and Feng *et al.* (2019), confirming preservation of the Mo–C and Ti–C framework.^75–78^ Both MXenes exhibited strong F and O signals, reflecting surface terminations introduced during HF etching and subsequent ambient oxidation, in line with Alhabeb *et al.* (2017).^28^ The higher carbon content is attributed to the direct adherence of both titanium and molybdenum MAX phase, m-MXene, and d-MXene powders to the carbon tape during SEM and EDS analyses. The slightly elevated O and F contents, compared to some previous reports, can be ascribed to the optimized etching ratios employed, which promote enhanced termination coverage and surface uniformity. These findings not only align with earlier studies but also set new benchmarks, highlighting how the etching chemistry critically governs the surface termination density and stability of MXenes.

### 3.4 Raman analysis of Mo_2_Ga_2_C and Ti_3_AlC_2_ systems

**Fig. 7:**
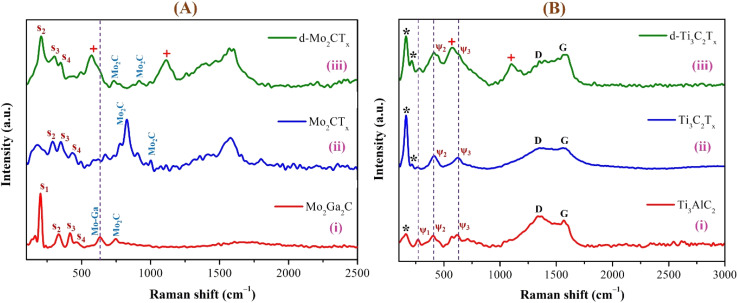
Raman spectra of the (A) Mo_2_Ga_2_C and (B) Ti_3_AlC_2_ systems, shown for their (i) MAX phase, (ii) m-MXene, and (iii) d-MXene. For Mo_2_Ga_2_C, *S*_1_, *S*_2_, and *S*_3_ peaks correspond to the shear (*E*_2g_) and longitudinal (*A*_1g_) vibrational modes of Mo and Ga atoms along the *c*-axis; the diminution of Ga-related peaks after etching is marked with dashed lines. In Ti_3_AlC_2_, *ψ*_1_, *ψ*_2_, and *ψ*_3_ peaks correspond to *E*_2g_ and *A*_1g_ modes of Ti and Al. The *ψ*_1_ disappears after etching and the peak at 156.3 cm^−1^ (“*”) denotes out-of-plane Ti/C vibrations.

The Raman spectra of Mo_2_Ga_2_C and Ti_3_AlC_2_ systems of MAX phase, m-MXene, and d-MXene are presented in Fig. 7. The Raman peaks were observed at different stages of the material transformation, highlighting structural and vibrational changes during etching and delamination. The Raman spectrum of the Mo_2_Ga_2_C MAX phase (Fig. 7A(i), red curve) exhibited distinct peaks at 201.5, 331.8, 419.8, 633.1, and 748.4 cm^−1^. The Raman bands at 201.5, 331.8, and 419.8 cm^−1^ labeled as *S*_1_, *S*_2_, and *S*_3_ correspond to the characteristic shear (E_2g_) and longitudinal (A_1g_) vibrational modes of Mo and Ga atoms along the *c*-axis in the layered MAX phase structure.^79,80^ Specifically, the *S*_1_ peak is associated with the vibrations of Ga atoms.^81^ The peaks at 633.1 and 748.4 cm^−1^ are attributed to Mo–Ga and Mo_2_C vibrations within an octahedral coordination. Upon etching to form Mo_2_CT_*x*_ m-MXenes (Fig. 7A(ii), blue curve), significant changes were observed in the Raman spectrum. A sharp, high-intensity peak appeared at 827.7 cm^−1^, while the peaks at 201.5 and 633.1 cm^−1^ corresponding to the vibrations of Ga and Mo–Ga in the MAX phase were significantly reduced, indicating the successful removal of Ga layers and the formation of Mo_2_CT_*x*_ MXene. The peaks at 827.7 and 998.1 cm^−1^ are characteristic of the *E*_2g_ and *A*_1g_ vibrations of the Mo_2_C layer in the MXene structure.^82–84^ Low-intensity peaks at 289.3, 350.2, and 432.5 cm^−1^ (labeled as *S*_2_, *S*_3_, and *S*_4_ in Fig. 7A(ii)) correspond to out-of-plane Mo and C vibrations influenced by surface terminations (–F, –O, or –OH groups) introduced during the etching process.^85^ For delaminated Mo_2_CT_*x*_ MXenes (Fig. 7A(iii), green curve), further spectral changes confirmed successful delamination. The increased intensity and sharpness of the peak at 207.2 cm^−1^ (labeled as *S*_2_ in Fig. 7A(iii)) are attributed to the formation of surface oxide on the Mo_2_CT_*x*_ MXene surface.^86^ Additional peaks at 298.5 and 348.4 cm^−1^ (labeled as *S*_3_ and *S*_4_ in Fig. 7A(iii)) were attributed to Mo–C vibrations influenced by intercalated Li^+^ ions or structural rearrangements.^80^ New peaks at 574.6 and 1110.2 cm^−1^ were identified as artifacts from the glass microscope slide (marked with “+” in Fig. 7A(iii)) used for MXene sample preparation as the substrate.^87,88^ The Raman peaks at 207.2, 298.5, and 348.4 cm^−1^ exhibited slight shifts compared to the corresponding peaks at 289.3, 350.2, and 432.5 cm^−1^ of m-MXenes, probably due to interference from the glass substrate peaks (574.6 and 1110.2 cm^−1^). Similarly, the characteristic peaks of Mo_2_CT_*x*_ MXenes at 827.7 and 998.1 cm^−1^ shifted slightly to 729.4 and 912.6 cm^−1^, which also attributed to the glass substrate. The peaks at 729.4 and 912.6 cm^−1^ represent characteristic Mo_2_C vibrations, with their reduced intensity further confirming the successful delamination of the MXene.

The Raman spectrum of the Ti_3_AlC_2_ MAX phase exhibits distinct peaks at 156.3, 270.7, 403.4, 617.2, 1346.1, and 1569.3 cm^−1^ (Fig. 7B(i), red curve). The characteristic peaks of the layered MAX structure at 270.7, 403.4, and 617.2 cm^−1^ are labeled as *ψ*_1_, *ψ*_2_, and *ψ*_3_, respectively, and correspond to the *E*_2g_ and *A*_1g_ vibrational modes of Ti and Al atoms.^89–92^ The *ψ*_1_ peak is specifically associated with the vibrations of Al atoms. The peak at 156.3 cm^−1^ marked with “*” is attributed to the out-of-plane vibrations of Ti and C atoms.^93^ Raman bands at 1346.1 and 1569.3 cm^−1^ are associated with sp^2^-hybridized carbon, indicating the presence of carbon-based vibrations in the structure.^94^ Upon etching to produce m-MXenes, significant changes were observed in the Raman spectrum (Fig. 7B(ii), blue curve). The disappearance of the *ψ*_1_ peak in the MXene spectrum correlates with the successful removal of Al layers and the formation of the MXene structure. Low-intensity peaks at 408.8 and 624.2 cm^−1^ correspond to Ti–C vibrational modes influenced by surface terminations such as –F, –O, or –OH groups.^86,93,95,96^ A sharp high-intensity peak at 163.9 cm^−1^ and a low-intensity peak at 214.7 cm^−1^, marked with “*”, are attributed to the formation of oxide on the Ti_3_C_2_ surface^86,97,98^ and the out-of-plane vibrations of Ti–C atoms, respectively.^99,100^ The D and G bands, located at 1337.4 and 1578.5 cm^−1^, respectively, indicate the presence of disordered and graphitic carbon within the material.^101,102^ Further changes in the Raman spectrum of delaminated Ti_3_C_2_T_*x*_ MXenes confirmed the successful delamination. The d-MXene spectrum displayed peaks at 163.9, 216.5, 408.1, 578.2, 638.4, and 1102.1 cm^−1^ (Fig. 7B(iii), green curve). The peaks at 163.9 and 216.5 cm^−1^ are similar to those observed in m-MXenes correspond to the surface-oxidized Ti_3_C_2_ peak and out-of-plane vibrations of Ti–C atoms, respectively (marked with “*”). However, the intensity of the 163.9 cm^−1^ peak was reduced in the delaminated spectrum. The peaks at 408.1 and 638.4 cm^−1^ are associated with Ti–C vibrations, with their increased intensity probably influenced by interactions with intercalated ions or functional groups.^103^ Conversely, the peaks at 578.2 and 1102.1 cm^−1^ are attributed to artifacts from the glass microscope slide (marked with “+”) used for Raman sample preparation.^87,88^ The slight shift observed in the 638.4 cm^−1^ peak compared to m-MXenes is probably due to Li^+^ ion intercalation and also the interference from the glass substrate. The D and G bands at 1346.8 and 1569.4 cm^−1^ remain prominent in the delaminated MXene spectrum, indicating that carbon-based vibrations are retained after delamination. The reduced intensity of the surface-oxidized peak at 163.9 cm^−1^ and the increased intensity of the characteristics peak of Ti_3_C_2_ vibration and G band in the d-MXene spectrum further confirm the successful delamination of Ti_3_C_2_T_*x*_ MXene. The *I*_D_/*I*_G_ ratio decreased for d-MXene (0.82) compared to the m-MXene (0.98) and the MAX phase (1.15), indicating the successful formation of the Ti_3_C_2_T_*x*_ d-MXene with a reduced structural disorder.

The Raman spectra distinctly captured the transformation from MAX phases to m-MXenes and d-MXenes, reflecting the structural and chemical modifications induced during the etching and delamination processes. In Mo_2_CT_*x*_, the disappearance of Ga-related peaks and the emergence of Mo–C vibrational modes at 729 and 912 cm^−1^, along with additional weak bands between 207 and 432 cm^−1^ associated with surface terminations, confirm the successful conversion. These observations align well with those reported by Bayhan *et al.* (2023) and Gupta *et al.* (2023), who identified similar Mo–C bands and termination-related features, indicating effective Ga removal and preservation of the carbide framework.^57,104^ Likewise, for Ti_3_C_2_T_*x*_, the spectra exhibit characteristic Ti–C modes at 163–217 and 408–638 cm^−1^, together with pronounced D (∼1340 cm^−1^) and G (∼1570 cm^−1^) bands, indicating sp^2^ carbon ordering. This spectral profile is consistent with earlier findings by Sarycheva and Gogotsi (2020), Ferrari and Robertson (2000), and Rajendran *et al.* (2022), who reported comparable Raman features in delaminated Ti-based MXenes.^92,95,99,101^ Overall, the Raman signatures of both MXenes substantiate the phase purity, structural integrity, and low defect density achieved through the present synthesis approach.

### 3.5 FTIR analysis of Mo_2_Ga_2_C and Ti_3_AlC_2_ systems

FTIR and Raman spectroscopies are complementary techniques widely used to study vibrational modes in materials. FTIR spectroscopy is particularly effective in detecting Raman-inactive vibrational modes, such as *E*_u_ and *A*_2u_, making it a valuable tool for characterizing surface terminations, such as chemical bonds, MXene-based organic hybrids, and hydrogen bonding.^105^ The FTIR spectra of Mo_2_Ga_2_C and Ti_3_AlC_2_ MAX phases, m-MXenes, and d-MXenes exhibit distinct features across two main regions. The surface functional group region (4000–1500 cm^−1^) reveals peaks associated with hydroxyl (–OH), oxygen (–O) and carbon bonds (C–H, C=O), while the fingerprint region (1500–400 cm^−1^) contains unique absorption peaks that identify molecular vibrations characteristic of each material's structure.

**Fig. 8:**
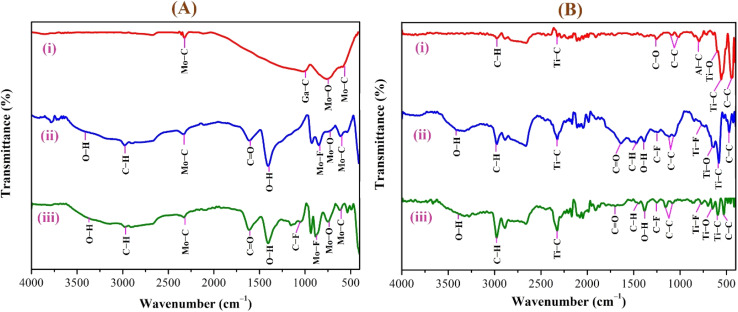
FTIR spectra of (A) Mo_2_Ga_2_C and (B) Ti_3_AlC_2_, showing transitions from (i) MAX to (ii) m-MXene and (iii) d-MXene. Characteristic vibration peaks of Mo–C/Ga–C and Ti–C/Al–C appear in the fingerprint region of both systems (Mo_2_Ga_2_C and Ti_3_AlC_2_). The Ga- and Al-related peaks diminish in the MXene spectra, and new peaks corresponding to surface functional group-related vibrations (–OH, –F, and =O) are pointed in the plotted graph.

For the Mo_2_Ga_2_C MAX phase (as shown in Fig. 8A(i), red curve), the Ga–C stretching peak at ∼1023 cm^−1^ is specific to gallium carbide bonds,^106^ Mo–O stretching at ∼762 cm^−1^ is linked to partial surface oxidation of molybdenum while Mo–C stretching and bending vibrations at ∼2322 cm^−1^ and ∼580 cm^−1^ are characteristic of molybdenum carbide in the MAX phase.^107,108^ The spectrum of the Mo_2_CT_*x*_ m-MXene (as shown in Fig. 8A(ii), blue curve) reveals O–H stretching vibrations between ∼3600 and ∼3200 cm^−1^, confirming the presence of hydroxyl surface terminations introduced during etching, and O–H bending at ∼1411 cm^−1^ further supports this functionalization.^99^ The C–H stretching at ∼2890 cm^−1^ reflects aliphatic hydrocarbon vibrations. The spectrum also shows C=O stretching at ∼1621 cm^−1^ reflecting carbonyl groups.^109,110^ Additionally, Mo–F stretching at ∼852 cm^−1^ indicates fluorine terminations from HF etching, Mo–O stretching at ∼733 cm^−1^ suggests molybdenum oxide species, the Mo–C stretching and bending at ∼2343 cm^−1^ and ∼610 cm^−1^ confirmed the presence of molybdenum carbide bonds.^109,110^ The spectrum of the Mo_2_CT_*x*_ d-MXene is similar to that of the m-MXene, with slight shifts in peaks due to delamination (Fig. 8A(iii), green curve). These include O–H bending at ∼1410 cm^−1^ and C–H stretching at ∼2901 cm^−1^. Additionally, Mo–F stretching at ∼883 cm^−1^ suggests increased fluorine terminations, while Mo–C stretching and bending at ∼2326 cm^−1^ and ∼619 cm^−1^ indicated the carbide framework's retention.

Similarly, the FTIR spectrum of the Ti_3_AlC_2_ MAX phase (Fig. 8B(i), red curve) confirms its structural integrity through distinct vibrational signatures. The C–H stretching at ∼2331 cm^−1^ indicates hydrocarbon interactions, while the C–O stretching at ∼1251 cm^−1^ suggests surface oxides or possible impurities. The Al–C stretching at ∼799 cm^−1^ underscores aluminum–carbon bonds, essential for the MAX phase structure.^111^ The Ti–O stretching at ∼599 cm^−1^ reveals partial surface oxidation of titanium, possibly due to environmental exposure, while the Ti–C stretching and bending at ∼2322 cm^−1^ and ∼559 cm^−1^ confirmed the presence of titanium carbide bonds, a defining feature of the MAX phase.^109,112^ Additionally, the C–C bending vibration at ∼445 cm^−1^ highlights in-plane deformation of carbon–carbon bonds, characteristic of the layered structure.^99,113^

The FTIR spectrum of the etched Ti_3_C_2_T_*x*_ m-MXene (Fig. 8B(ii), blue curve) shows significant functionalization and new surface terminations after etching process. The broad O–H stretching (3600–3200 cm^−1^) and O–H bending (∼1394 cm^−1^) confirmed the addition of –OH groups.^99^ The C–H stretching at ∼2892 cm^−1^ reflected hydrocarbon functionalities. C=O stretching at ∼1802 cm^−1^ indicated a carbonyl group.^109,110^ The C–F stretching at ∼1248 cm^−1^ and Ti–F stretching at ∼764 cm^−1^ confirmed fluorination from the etching solution, while Ti–O stretching at ∼654 cm^−1^ and Ti–C stretching and bending at ∼2323 cm^−1^ and ∼584 cm^−1^ demonstrated retention of titanium oxide and carbide bonds.^109,112^ The Ti_3_C_2_T_*x*_ d-MXene spectrum (Fig. 8B(iii), green curve) resembles that of the m-MXene, with peak shifts due to delamination. These include O–H bending at ∼1410 cm^−1^ and C–H stretching at ∼2901 cm^−1^, reflecting altered chemical environments. Enhanced C–F stretching at ∼1252 cm^−1^ and Ti–F stretching at ∼773 cm^−1^ highlighted fluorination, while Ti–O stretching at ∼652 cm^−1^ and Ti–C stretching and bending at ∼2327 cm^−1^ and ∼598 cm^−1^ confirmed structural stability. A C–C bending vibration in the range of 550−450 cm^−1^ is observed in the Ti_3_AlC_2_ MAX phase, m-MXene, and d-MXene, within the fingerprint region.^99,113^ However, this vibration corresponds to a weak *A*_2u_ (C–C) IR mode, making it less significant for FTIR-based identification of Ti_3_AlC_2_. The FTIR analysis revealed the detailed surface chemistry of both Mo_2_CT_*x*_ and Ti_3_C_2_T_*x*_ MXenes following etching and delamination processes.^105^

This is evidenced by the appearance of broad –OH peaks, which are absent in the MAX phase but become prominent in the m-MXene and d-MXene, indicating surface functionalization. For Mo_2_CT_*x*_, distinct O–H, C–H, C=O, and Mo–F absorption bands confirmed the presence of hydroxyl, carboxyl, and fluoride terminations, while the preserved Mo–C vibrations indicate retention of the carbide framework. These features are consistent with DFT predictions and spectral assignments reported by Parker *et al.* (2024), reflecting the complex and overlapping contributions of mixed surface terminations.^105^ Similarly, Ti_3_C_2_T_*x*_ exhibits characteristic O–H, C=O, Ti–F, and backbone Ti–C vibrational modes, in agreement with both theoretical and experimental analyses by Hu *et al.* (2015) and Parker *et al.* (2024).^99,105^ Collectively, these studies establish a robust framework for interpreting MXene FTIR signatures. The strong correspondence between experimental spectra and DFT-predicted vibrational modes (Table 2) confirms effective delamination, functionalization, and structural integrity across Mo- and Ti-based MXenes, thereby validating the surface chemistry and phase preservation crucial for advanced MXene applications. Moreover, the FTIR vibrational modes of Mo_2_CT_*x*_ and Ti_3_C_2_T_*x*_ (Table 2) demonstrate strong alignment between empirically observed peaks and DFT-predicted modes for surface terminations such as –F_2_ and –O_2_, further supporting the presence of these functional groups on the MXene surfaces.

**Table 2:** FTIR vibration modes of Mo_2_CT_*x*_ and Ti_3_C_2_T_*x*_ MXenes: empirically determined^114^*vs.* DFT-predicted for –F_2_ and –O_2_ terminations (T_*x*_)^105^^a^

MXene type	MXene vibration modes			
	M–C ∼350−450 cm^−1^	C–C ∼400−500 cm^−1^	M–O ∼500−650 cm^−1^	M–F ∼700−750 cm^−1^
Mo_2_CT_*x*_	(Mo–C)^99,115,116^	(C–C)^114,117–119^	(Mo–O)^99,115,116^	(Mo–F)^116,120^
	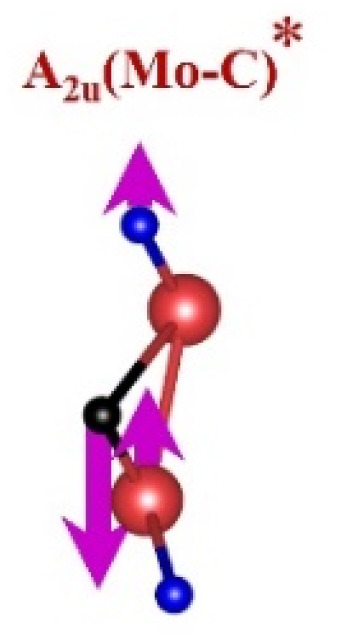	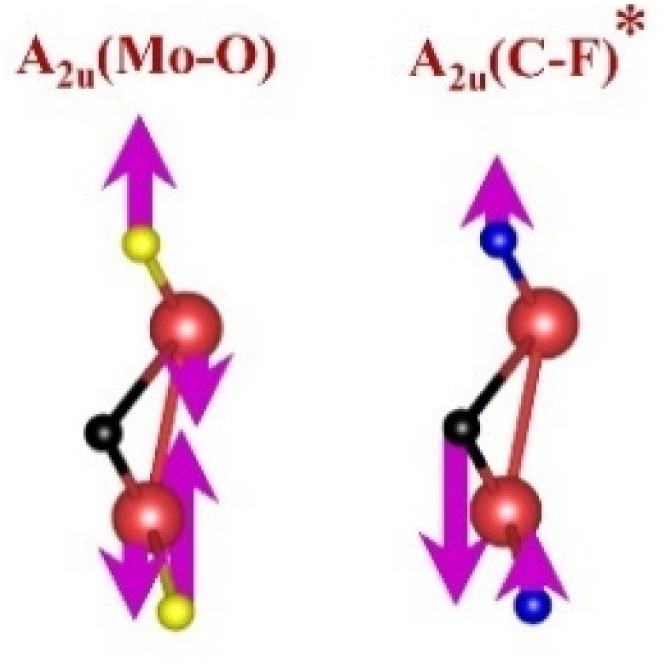	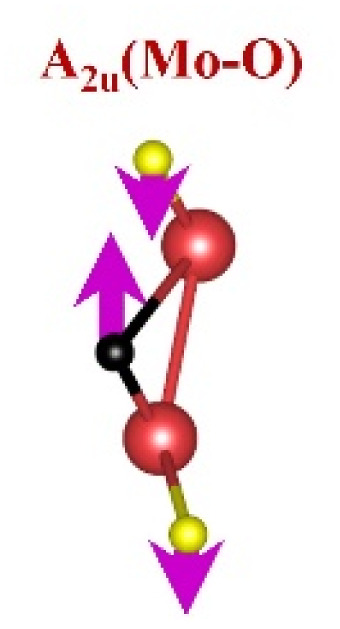	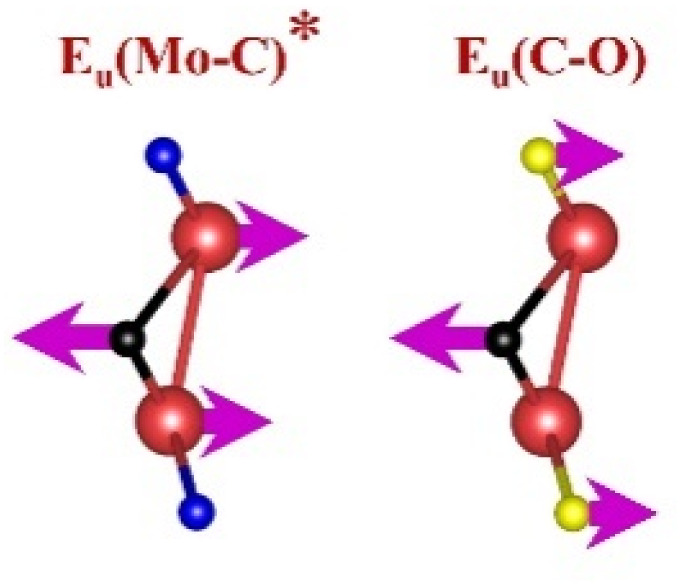
Ti_3_C_2_T_*x*_	(Ti–C)^109,110^	(C–C)^114,117–119^	(Ti–O)^109,110^	(Ti–F)^118,120^
	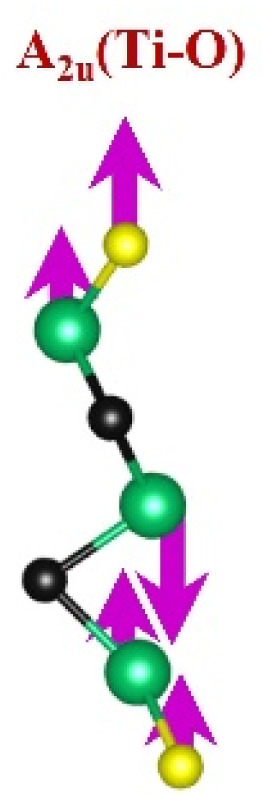	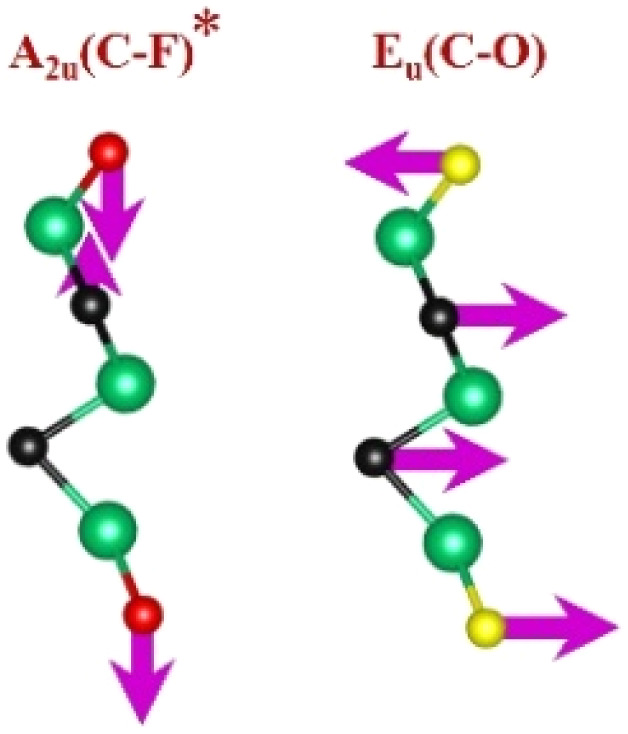	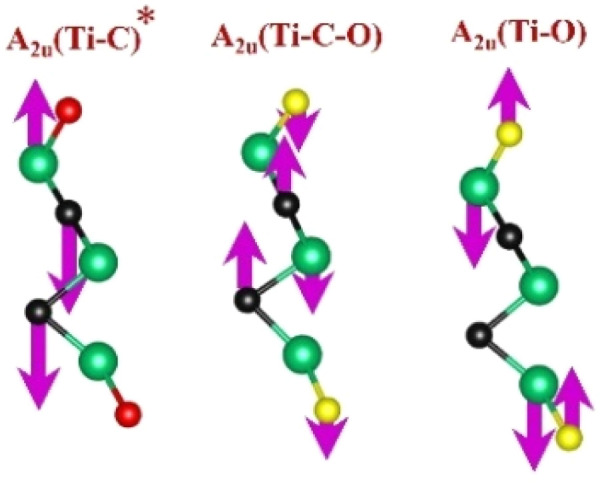	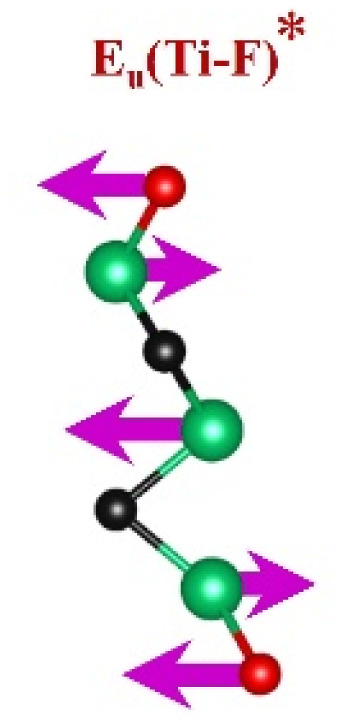

a Footnotes: in the table, empirical terminations are presented in parentheses, while DFT-predicted terminations with –F are marked with “*”, and terminations with =O remain unmarked. Discrepancies between DFT predictions and empirical data arise from the use of a single-termination model and the inherently broad range of empirical FTIR data. All structural visualizations were created using the VESTA software.

## 4 Limitations of the study

While this study provides the first systematic comparative synthesis of Mo_2_CT_*x*_ and Ti_3_C_2_T_*x*_ MXenes under identical processing conditions, several limitations should be noted. First, the investigation was restricted to only two MXene systems (Mo- and Ti-based), and the conclusions may not directly extend to other emerging MXene families. Second, the structural and morphological characterizations were comprehensive. However, quantitative surface chemistry (*e.g.*, XPS depth profiling) and computational modeling (*e.g.*, DFT-based electronic structure calculations) were not included, which would have further validated the interpretations of the UV-Vis, FTIR, and Raman features. Third, only short-term oxidation stability (up to 32 days) was observed, despite the importance of long-term stability for practical applications. Finally, the study was limited to laboratory-scale synthesis; thus, the scalability of the optimized etching protocols to industrial levels remains yet to be demonstrated. These limitations define the scope of our work while also pointing to clear directions for future research aimed at extending the comparative framework to other MXene families, integrating advanced surface/electronic characterizations, and assessing long-term performance in device-relevant environments.

## 5 Conclusion

This study demonstrates a comparative scalable synthesis strategy of both multilayer and delaminated Mo_2_CT_*x*_ and Ti_3_C_2_T_*x*_ MXenes, by tailored acid etching and LiCl-based delamination without organic intercalants or ultrasonication. This unified protocol contrasts with conventional *in situ* HF and concentrated HF approaches by yielding enhanced flake quality, scalable yields, and reproducible control of MXene morphology and surface chemistry. Key findings including significant interlayer expansion (XRD shifts: 9.81° → 8.03° for Mo_2_CT_*x*_, 9.51° → 8.80° for Ti_3_C_2_T_*x*_), lateral sheet sizes of 0.5–1 µm (Mo_2_CT_*x*_) and 2–5 µm (Ti_3_C_2_T_*x*_), and distinct UV-Vis absorption features confirmed the strong electronic transitions and high-quality delamination. HR-SEM and EDS analyses confirmed robust morphology and elemental composition, while spectroscopic analysis confirms delamination and structural integrity. This work offers a direct, rigorous comparison of Mo- and Ti-based MXenes under identical conditions, showcasing a facile, safe, scalable route to high-quality MXenes with well-preserved carbide frameworks and surface terminations (–OH, –F, and =O). These results provide critical comparative data and clearly demonstrated how etchant formulation and delamination strategies influence the MXene properties. Although no application-specific measurements are included, the detailed structural benchmarks established here serve as a foundation for future research into energy, sensing, and filtration technologies utilizing MXenes.

## Author contributions

Conceptualization: V. M. and A. K. S.; methodology, validation, formal analysis, data curation, software, writing – original draft preparation: V. M.; resources, review and editing: R. A., S. A., and S. H. M.; resources, supervision, project administration, funding acquisition, writing – review & editing, validation: A. K. S. all authors have read and agreed to the published version of the manuscript.

## Conflicts of interest

The authors declare no conflict of interest.

## Supplementary Material

NA-OLF-D5NA00669D-s001

## Data Availability

We have provided all the obtained data in the manuscript. Supplementary information: includes photographs of MAXphase impurity removal, delaminated MXene dispersion, and oxidation stability of d-MXene, along with histogram plots of the lateral sheet size distribution for Mo2CTx and Ti3C2Tx MXenes. See DOI: https://doi.org/10.1039/d5na00669d.
