# Supplementary material for: Tailored synthesis and morphological analysis of Mo2CTx and Ti3C2Tx MXenes: a study on multilayered and delaminated architectures
Source: Nanoscale Adv. 2025 Nov 3;7(24):8122–37. doi: 10.1039/d5na00669d (PMC12628277; doi:10.1039/d5na00669d)
Supplement: NA-007-D5NA00669D-s001 [file NA-007-D5NA00669D-s001.pdf]

Supplementary Information (SI) for Nanoscale Advances.
This journal is © The Royal Society of Chemistry 2025

SUPPORTING INFORMATION

Tailored Synthesis and Morphological Analysis of Mo_2CT_x and $Ti_3C_2T_x$ MXenes: A Study on Multilayered and Delaminated Architectures

Vasanth Magesh[1,a], Raji Atchudan[2,a], Sandeep Arya[3], Surendra H. Mahadevegowda[4], and Ashok K. Sundramoorthy[*,1]*

[1]Department of Prosthodontics and Materials Science, Saveetha Dental College and Hospitals, Saveetha Institute of Medical and Technical Sciences, Chennai, 600077, Tamil Nadu, India

[2]School of Chemical Engineering, Yeungnam University, Gyeongsan 38541, Republic of Korea

[3]Department of Physics, University of Jammu, Jammu 180006, Jammu and Kashmir, India

[4]Department of Chemistry, School of Sciences, National Institute of Technology Andhra Pradesh, Tadepalligudem 534101, Andhra Pradesh, India

* Corresponding author.

[a] These authors equally contributed.

E-mail addresses: ashok.sundramoorthy@gmail.com

(A.K. Sundramoorthy).

Photographs of MAX phase washing, d-MXene dispersion, and d-MXene oxidation stability:

To remove impurities from the MAX phase, 1 g each of Mo_2Ga_2C and Ti_3AlC_2 MAX phase powders were separately immersed in 9 M HCl. The mixtures were briefly stirred with a glass rod and left undisturbed for 24 h. In the Ti_3AlC_2 mixture, air bubbles (likely hydrogen gas [$Al_{(s)}$+ $HCl_{(l)}$ → $AlCl_3$+$H_{2(g)}$]) began to emerge from the powder within 2 min, and 2 h later the powder settled, leaving a pink hue in the supernatant. After 24 h, the supernatant completely turned pinkish-purple (**Fig. S1A**). In contrast, no air bubbles were observed in the Mo_2GaC mixture. After 2 h, the powder settled and the supernatant showed a yellow hue, which deepened to a golden-yellow after 24 h (**Fig. S1B**).

The MAX phase powder treated with 9 M HCl was collected using vacuum filtration and dried in a vacuum oven at 80˚C overnight. The dried Mo_2Ga_2C MAX phase appeared as a greenish-grey powder with a flour-like texture (**Fig. S2A**). In contrast, the dried Ti_3AlC_2 MAX phase had a blackish-grey appearance with a texture resembling fine black sand (**Fig. S2B**).

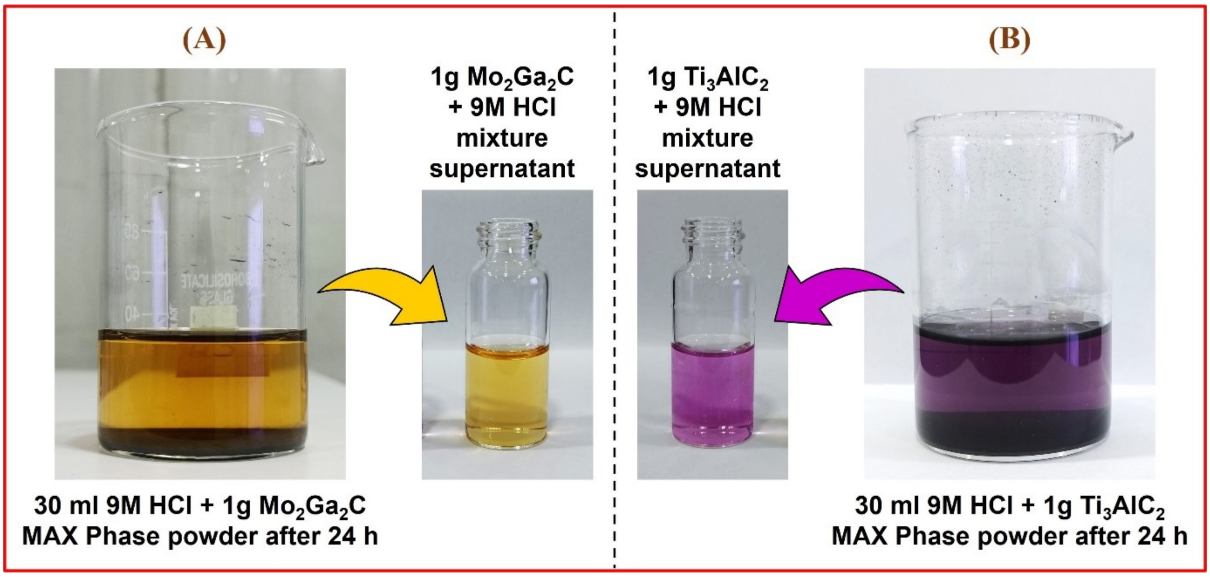

Figure S1. Washing of Mo_2Ga_2C **(A)** and Ti_3AlC_2 **(B)** MAX phase powders with 9 M HCl to remove residual or unreacted precursor materials, such as Mo, Ga, and C in Mo_2Ga_2C, and Ti, Al, and C in Ti_3AlC_2. The washing process effectively dissolves these impurities, as evidenced by the distinct supernatant colors: a golden yellow hue for Mo_2Ga_2C and a pinkish-purple tint for Ti_3AlC_2. These colors indicate the presence of dissolved metal and carbide species reacting with HCl, confirming the removal of unreacted elements. The remaining solid powders predominantly consist of the purified MAX phase, now prepared for subsequent MXene synthesis steps. This washing procedure ensures high purity and consistency of starting materials for reliable etching and delamination.

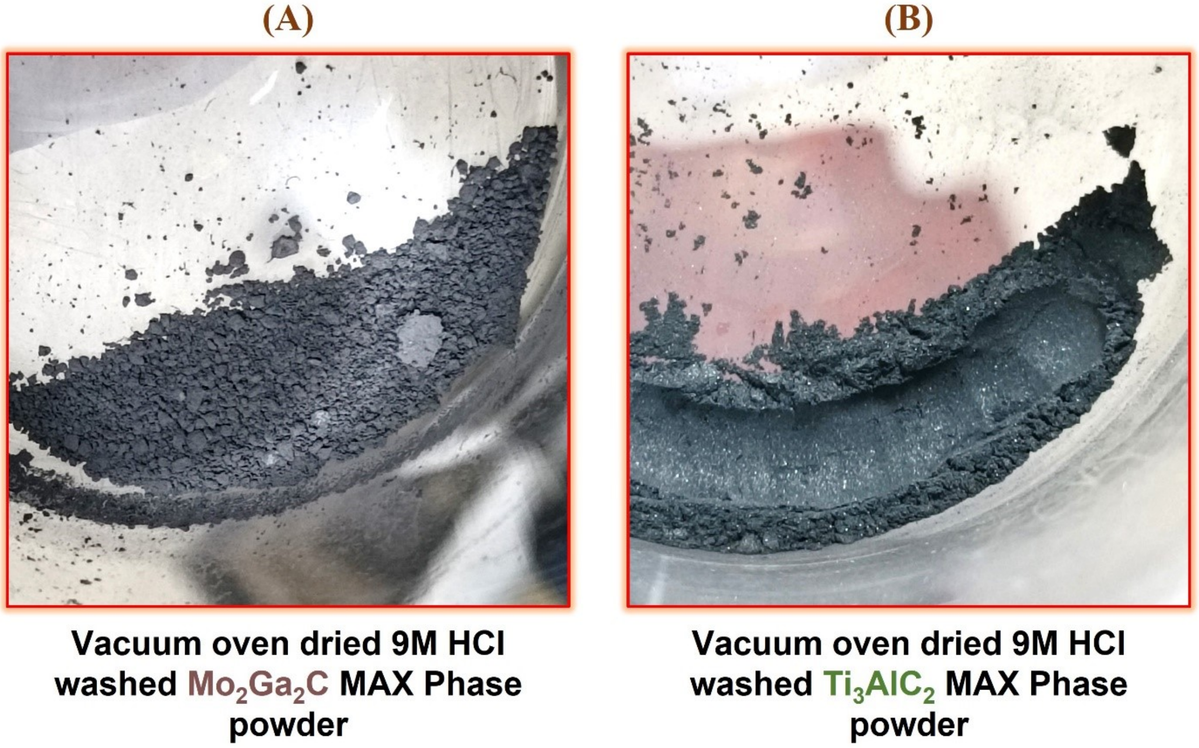

Figure S2. Photographs of vacuum-dried MAX phase powders following treatment with 9 M HCl: **(A)** Mo_2Ga_2C and **(B)** Ti_3AlC_2. The Mo_2Ga_2C MAX phase appears as a greenish-grey powder with a soft, flour-like texture, indicating fine particle morphology and good homogeneity after acid washing and drying. In contrast, the Ti_2AlC_2 MAX phase exhibits a blackish-grey color with a texture resembling fine black sand, suggesting slightly coarser granularity and denser packing. These distinct visual and tactile differences reflect the

influence of precursor composition and acid treatment on the physical characteristics of each MAX phase prior to subsequent MXene synthesis steps.

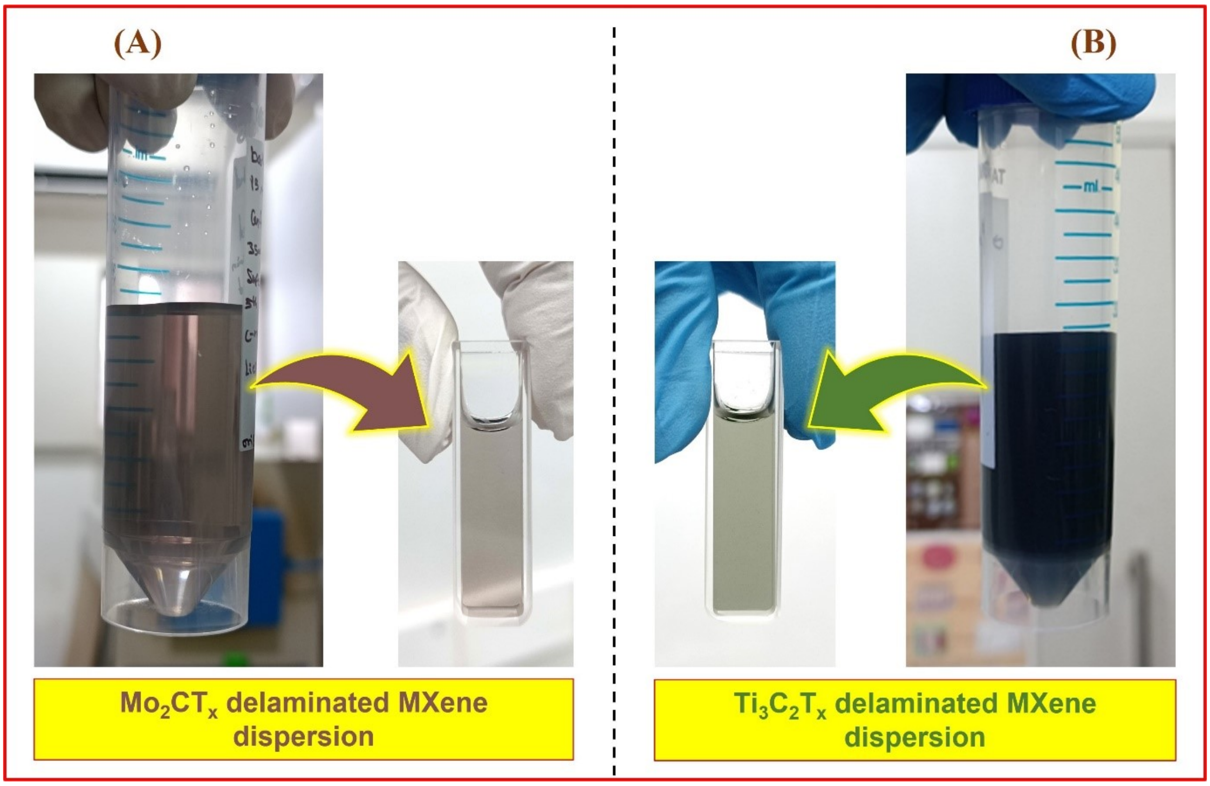

Figure S3. Photographs of **(A)** Mo_2CT_x and **(B)** $Ti_3C_2T_x$ d-MXene dispersions in DI water. The Mo_2CT_x d-MXene shows a transparent brown color in the centrifuge tube, which becomes lighter with a highly transparent brown tint upon transfer to a cuvette, indicating excellent colloidal stability and low concentration. In contrast, $Ti_3C_2T_x$ d-MXene forms a dark greenish-black suspension in the centrifuge tube that shifts to a transparent green upon dilution in a cuvette. These observations reveal visually distinct color characteristics for each MXene type, which are associated with their unique electronic structures and high-quality exfoliation and high dispersion in DI water.

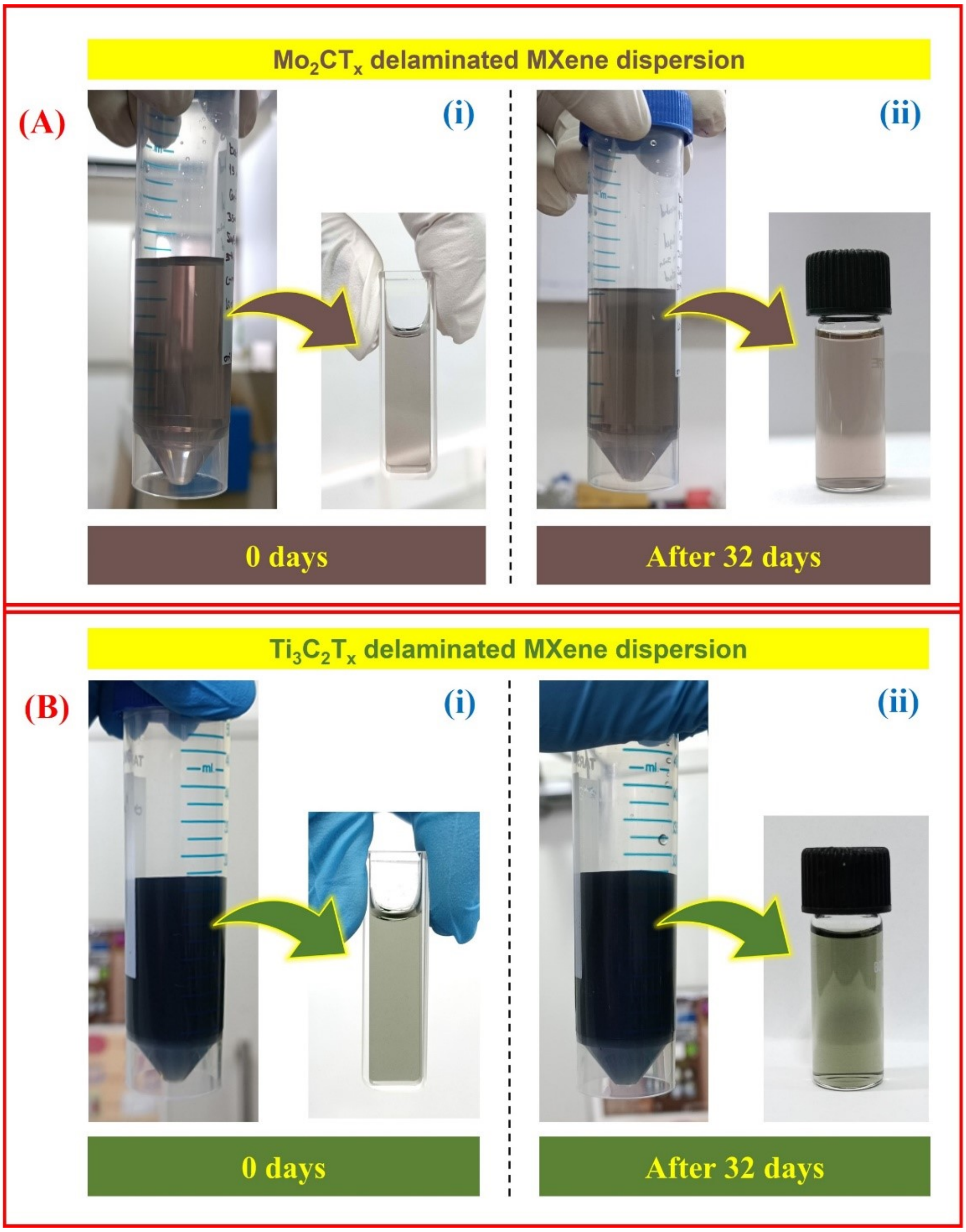

Figure S4. Oxidation stability of **(A)** Mo_2CT_x and **(B)** $Ti_3C_2T_x$ d-MXene dispersions in DI water, tracked over **(i)** 0 days and **(ii)** 32 days. Photographs show the freshly prepared d-MXene dispersions with high colloidal stability and no visible sedimentation or color change. After 32 days, both Mo_2CT_x and $Ti_3C_2T_x$ dispersions remain highly stable, maintaining their original color and only minimal sedimentation observed at the bottom of the tubes. These results indicate excellent oxidation resistance and prolonged dispersion stability for both MXene types under ambient conditions.

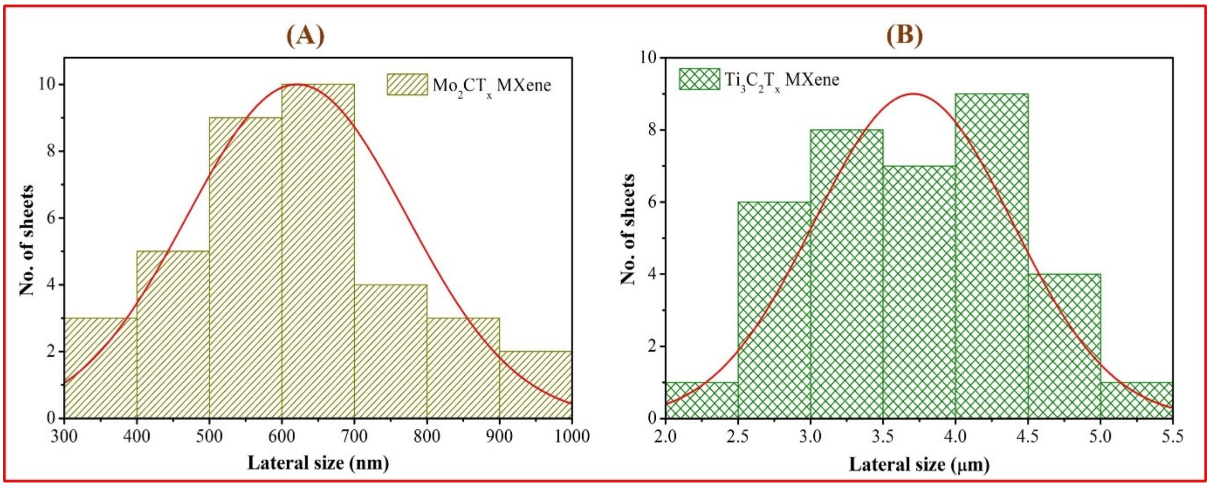

Figure S5. Lateral sheet size distribution of **(A)** Mo_2CT_x and **(B)** $Ti_3C_2T_x$ MXene. Histograms illustrate the measured lateral dimensions of exfoliated MXene sheets, as determined using ImageJ software from high-resolution SEM images. Origin software generated the plots and fitted the size distributions to a Normal (Gaussian) model. Analysis reveals a mean lateral sheet size of ~620 nm for Mo_2CT_x and ~3.7 μm for $Ti_3C_2T_x$. These results demonstrate a substantial difference in lateral sheet sizes between the Mo- and Ti-based MXenes under identical synthesis and delamination conditions, highlighting the influence of precursor chemistry on final MXene morphology.
